# Earlier diagnosis of intrahepatic cholestasis of pregnancy and adverse pregnancy outcomes

**DOI:** 10.1002/pmf2.70073

**Published:** 2025-07-16

**Authors:** Minhazur R. Sarker, Dana Canfield, Lauren Ferrara, Chelsea A. Debolt

**Affiliations:** ^1^ Department of Obstetrics Gynecology and Reproductive Science University of California San Diego San Diego California USA; ^2^ Department of Obstetrics Gynecology and Reproductive Science Mount Sinai Health System & Icahn School of Medicine at Mount Sinai New York New York USA; ^3^ Department of Obstetrics Gynecology and Reproductive Science New York City Health and Hospitals, Elmhurst Hospital Center Elmhurst New York USA

**Keywords:** bile acids, hepatobiliary disease, intrahepatic cholestasis, preterm birth, stillbirth

## Abstract

**Introduction:**

We aimed to determine whether pregnancies complicated by early diagnosis of cholestasis were associated with adverse maternal or neonatal outcomes.

**Methods:**

This is a retrospective cohort study of singleton, non‐anomalous live gestations complicated by cholestasis from 2005 to 2019. We compared rates of adverse outcomes in pregnancies complicated by early (< 32‐week gestational age) versus late (≥ 32‐week gestational age) diagnosis of cholestasis. Our primary outcome of interest was rates of spontaneous preterm birth. Secondary outcomes included rates of iatrogenic preterm birth, meconium‐stained amniotic fluid, cesarean delivery for non‐reassuring fetal heart tracing, and neonatal intensive care unit admission.

**Results:**

Of the 1247 pregnancies complicated by cholestasis, 241 (19.3%) had early diagnosis and 1006 (80.7%) had late diagnosis. After adjusting for confounders including peak total bile acid levels, earlier diagnosis of cholestasis remained associated with spontaneous preterm birth (OR 1.81; 95% CI 1.11–2.95), iatrogenic preterm birth (OR 1.59; 95% CI 1.12–2.67), and NICU admission (OR 1.43; 95% CI 1.04–1.95). A sub‐analysis to compare outcomes with severe cholestasis (peak total bile acids ≥ 40 µmol/L) showed that early diagnosis of severe cholestasis was also associated with spontaneous preterm labor (OR 2.41; 95% CI 1.34–4.34), iatrogenic preterm birth (OR 1.67; 95% CI 1.05–2.67), and NICU admission (OR 1.66; 95% CI 1.06–2.61).

**Conclusion:**

Findings suggest that earlier diagnosis of cholestasis is associated with adverse outcomes and that this is not entirely driven by peak total bile acid levels.

## 1 INTRODUCTION

Intrahepatic cholestasis of pregnancy (ICP) is the most common hepatobiliary disease diagnosed in pregnancy [1]. Typically, ICP occurs in the third trimester with pruritis of the palms and soles worsening at night and an associated elevation in serum total bile acids (TBAs) [2]. Although maternal complications are rare, case reports have documented various complications primarily associated with fat‐soluble vitamin deficiency [3, 4, 5]. More clinically concerning are the adverse fetal outcomes, including stillbirth, spontaneous preterm birth, iatrogenic preterm birth, meconium‐stained amniotic fluid, and neonatal respiratory distress syndrome [6, 7, 8, 9, 10]. Management of ICP is generally guided by the peak TBA level, a marker for disease severity. Recently, studies have shown a positive association between TBA levels and adverse outcomes [6, 7, 9, 10, 11, 12]. Specifically, outcomes appear to be worse with severe ICP (TBA ≥ 40 µmol/L).

Furthermore, although a prior study has shown that bile acids may increase throughout pregnancy, whether trending TBA levels improve outcomes remains to be seen [13]. Consequently, there are no current guidelines surrounding whether TBA levels should be repeated, and if so, when this should occur. Given the lack of a management paradigm, diagnosis of ICP prior to late third trimester has limited evidence‐based approaches. Currently, management is not altered by gestational age at diagnosis of ICP. A prior study revealed that earlier diagnosis of ICP was associated with higher TBA levels and increased rates of meconium‐stained amniotic fluid, fetal distress, and neonatal asphyxia [14]. A second study also found that diagnosis of earlier diagnosis of ICP was associated with adverse outcomes including preterm labor [15]. However, both of these studies had relatively limited cohort sizes and predate more recent research, which has provided more thorough characterization of adverse pregnancy outcomes associated with ICP.

Our objective was to determine if there is an association between early diagnosis of ICP and adverse outcomes inclusive of outcomes more recently characterized in contemporary studies. We hypothesized that early ICP would be associated with more adverse outcomes than late ICP owing to the earlier exposure to elevated bile acids, a marker for cholestasis. Additionally, we aimed to determine how the associations differ specifically in cases of severe ICP and whether early severe ICP warrants additional caution.

## 2 MATERIALS AND METHODS

We conducted a retrospective cohort study of non‐anomalous, singleton, live gestations complicated by ICP that received prenatal care and were delivered at a single hospital center from 2005 to 2019. Our institution is a safety net city hospital that does not serve as a referral or transfer center for cholestasis. Likely due to the primarily Hispanic and South Asian population, we have a 7%–10% prevalence of ICP, which is markedly higher than the typical incidence of 0.3%–0.5% [16, 17]. Our obstetrical providers often screen for symptomatic ICP during prenatal visits, resulting in early diagnosis. Upon diagnosis (TBA > 10 µmol/L), patients are started on ursodeoxycholic acid at 300 mg twice daily and twice weekly fetal non‐stress testing with amniotic fluid assessments. Additionally, although no established or validated protocols exist for TBA trending, we empirically trend TBA levels every 3–4 weeks until medically indicated delivery would be performed. At our institution, we deliver all cholestasis patients at 37 weeks and as early as 36 weeks for TBA > 100 µmol/L.

We compared rates of adverse outcomes in pregnancies complicated by early (< 32‐week gestation) versus late (≥ 32‐week gestation) diagnosis of ICP. Despite prior studies defining early onset ICP with varying cutoffs, no consensus for early diagnosis has been determined [14, 15, 18]. Further studies and management guidelines have favored planned 36–37‐week deliveries for pregnancies complicated by ICP such that we opted for 32‐week gestational age cutoff to define early ICP and performed a sensitivity analysis for very early ICP defined as less than 28‐week diagnosis [2, 19]. We excluded patients with underlying hepatobiliary pathology (history of hepatitis, acute fatty liver of pregnancy, cirrhosis, hepatocellular carcinoma, or primary biliary cirrhosis) and patients under the age of 18. A sub‐analysis was performed to compare rates of adverse outcomes with early versus late diagnosis of severe cholestasis (peak TBAs ≥ 40 µmol/L). Data were retrospectively abstracted from maternal and neonatal charts within the electronic medical record and then stored in a Research Electronic Data Capture (REDCap) database.

The primary outcome of interest was rate of spontaneous preterm birth defined as onset of labor or rupture of membranes and resultant delivery prior to 37 weeks’ gestation. The secondary outcomes included rates of iatrogenic preterm birth defined as induction of labor or scheduled cesarean delivery (CD) prior to 37 weeks, meconium‐stained amniotic fluid at any time during labor or delivery, CD for non‐reassuring fetal heart tracing (NRFHT), umbilical artery pH less than 7.20, and neonatal ICU (NICU) admission.

Chi‐square, Fisher exact, Student's *t*, and Mann–Whitney *U* tests were used for univariable analysis based on results distribution and sample sizes as statistically appropriate. Multivariable regression tests were used to determine the strengths of association between early ICP and adverse outcomes. Multivariable analysis was adjusted for peak TBA level, history of preterm birth, and ursodeoxycholic acid treatment. All statistical analyses were performed on STATA IC Version 15. A *p* value of less than 0.05 and 95% CI not crossing 1.00 indicated statistical significance. A waiver of informed consent was obtained from the Institutional Review Board since our study posed minimal risk to the patients.

## 3 RESULTS

**FIGURE 1:**
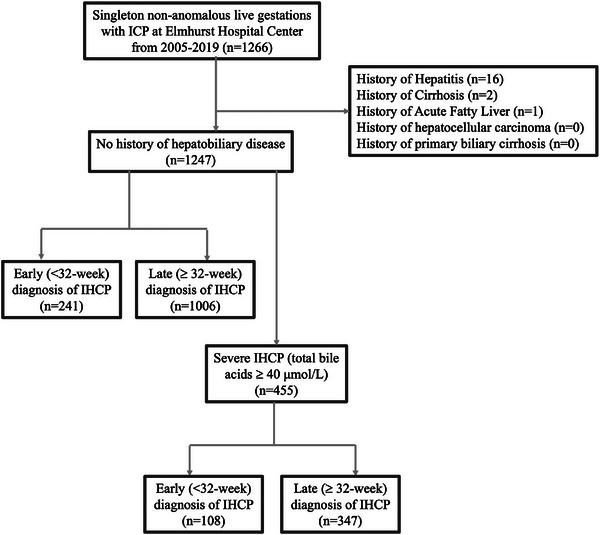
Study population flowchart highlighting final cohort breakdown for primary analysis and severe ICP sub‐analysis.

During our study period, there were 1247 singleton, non‐anomalous, live gestations complicated by ICP of which 241 (19.3%) had early diagnosis and 1006 (80.7%) had late diagnosis of ICP (Figure 1). Pregnancies with early diagnosis of ICP had higher peak TBA levels, increasing prevalence of higher ICP severity, higher rates of prior cholestasis, higher rates of prior preterm birth, higher incidence of ursodeoxycholic acid treatment, and were delivered at an earlier gestational age (all *p* < 0.01) (Table 1). There were no statistically significant differences noted between early and late diagnosis of ICP and maternal age, ethnicity, comorbidities, prior CD, prior intrauterine fetal demise (IUFD), or delivery mode (Table 1).

**TABLE 1:** Maternal baseline demographics and characteristics among early versus late ICP.

	Early ICP (*n* = 241)	Late ICP (*n* = 1006)	*p* value
Gestational age at diagnosis	27.21 ± 5.52	36.12 ± 2.07	<0.01
Peak Total Bile Acids	53.38 ± 50.57	43.05 ± 41.29	<0.01
ICP Severity Cohort			0.01
Mild ICP (TBA 10–19 µmol/L)	60 (24.90)	265 (26.34)	
Moderate ICP (TBA 20–29 µmol/L)	73 (30.29)	390 (38.8)	
Severe ICP (TBA 40–99 µmol/L)	74 (30.71)	257 (25.55)	
Very Severe ICP (TBA ≥ 100 µmol/L)	34 (14.11)	94 (9.34)	
Age	29.45 ± 5.95	28.50 ± 5.84	0.95
Ethnicity			0.05
Hispanic	181 (75.73)	833 (83.38)	
Bengali	30 (12.55)	68 (6.81)	
European	1 (0.42)	5 (0.50)	
African	2 (0.84)	4 (0.40)	
Other/Unknown	9 (3.77)	27 (2.70)	
Southeast Asian	16 (6.69)	62 (6.21)	
Parity, [interquartile range]	1 [0,2]	1 [0,2]	0.15
History of cholestasis	54 (22.50)	124 (12.34)	< 0.01
Prior preterm birth	28 (11.62)	67 (6.66)	< 0.01
Prior cesarean section	38 (16.10)	119 (12.34)	0.13
Prior Stillbirth	5 (5.21)	6 (2.15)	0.13
Pre‐gestational diabetes	8 (3.32)	18 (1.79)	0.14
Gestational diabetes	43 (17.84)	165 (16.40)	0.59
Pregnancy Induced Hypertension	17 (7.05)	88 (8.75)	0.40
Gestational age at delivery	36.36 ± 1.90	37.65 ± 1.28	< 0.01
Ursodeoxycholic acid Therapy	199 (82.57)	366 (36.53)	< 0.01
Delivery Mode			0.28
Vaginal	155 (64.32)	696 (69.18)	
Cesarean	82 (34.02)	290 (28.83)	

*Note*: All categorical variables reported as *n* (%). All continuous variables reported as mean ± SD unless listed otherwise.

**FIGURE 2:**
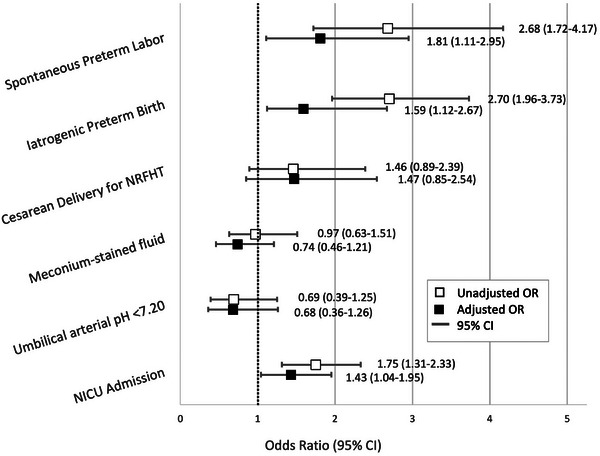
Multivariable logistic regression comparing early (*n* = 241) versus late (*n* = 1006) diagnosis of ICP and association with adverse outcomes. Results were adjusted for peak total bile acid, history of preterm birth, and ursodeoxycholic treatment. Upper and lower limit bars denote 95% confidence intervals and a confidence interval not crossing 1.00 indicates statistical significance (indicated by the dashed vertical line).

In the univariable analysis, earlier diagnosis of ICP was associated with spontaneous preterm birth (14.5% vs. 6.0%, *p* < 0.01), iatrogenic preterm birth (32.0% vs. 14.8%, *p* < 0.01), and NICU admission (50.7% vs. 37.0%, *p* < 0.01) (Table 2). After adjusting for confounders and statistically significant baseline differences including peak TBA levels, history of preterm birth, and ursodeoxycholic acid treatment, earlier diagnosis of ICP remained associated with spontaneous preterm birth (OR 1.81; 95% CI 1.11–2.95), iatrogenic preterm birth (OR 1.59; 95% CI 1.12–2.67), and NICU admission (OR 1.43; 95% CI 1.04–1.95) (Figure 2). Although there was a difference in the history of prior cholestasis, we previously showed that a history of cholestasis does not worsen outcomes in a subsequent pregnancy with cholestasis, and thus, we did not adjust for this in our regression models [20]. There was no statistically significant difference noted with early ICP and umbilical artery pH, CD for NRFHT, or meconium‐stained amniotic fluid (Table 2).

**TABLE 2:** Univariable analysis of early vs. late ICP diagnosis and adverse outcomes.

	Early ICP (*n* = 241)	Late ICP (*n* = 1006)	*p* value
Spontaneous preterm labor	35 (14.52)	60 (5.96)	< 0.01
Umbilical arterial pH < 7.20	14 (5.81)	82 (8.15)	0.22
Iatrogenic preterm birth	77 (31.95)	149 (14.81)	< 0.01
Cesarean delivery for non‐reassuring fetal heart rate tracing	23 (9.54)	68 (6.76)	0.14
Meconium‐stained fluid	27 (11.20)	116 (11.53)	0.89
Neonatal ICU admission	116 (50.66)	358 (37.02)	< 0.01

*Note*: All categorical variables reported as *n* (%).

**FIGURE 3:**
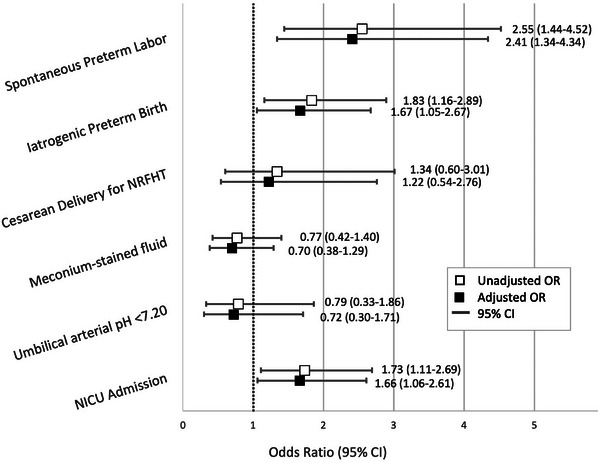
Multivariable logistic regression comparing early (*n* = 108) versus late (*n* = 347) diagnosis of severe ICP and association with adverse outcomes (sub‐analysis). Results were adjusted for peak total bile acid and history of preterm birth. Upper and lower limit bars denote 95% confidence intervals and a confidence interval not crossing 1.00 indicates statistical significance (indicated by the dashed vertical line).

Of the 1247 deliveries complicated by ICP, 455 (36.5%) had severe ICP of which 108 (23.7%) had early diagnosis and 347 (76.3%) had late diagnosis (Figure 1). Pregnancies with early diagnosis of severe ICP had higher rates of prior cholestasis, higher rates of prior preterm birth, higher incidence of ursodeoxycholic acid treatment, and were delivered at an earlier gestational age (all *p* < 0.01) (Table 3). In the univariable analysis, early diagnosis of severe ICP was likewise associated with spontaneous preterm birth (22.2% vs. 10.1%, *p* < 0.01), iatrogenic preterm birth (38.0% vs. 25.1%, *p* < 0.01), and NICU admission (57.6% vs. 43.9%, *p* = 0.01) (Table 4). In a multivariable regression adjusting for peak TBA levels and history of preterm birth, early diagnosis of severe ICP remained associated with spontaneous preterm birth (OR 2.41; 95% CI 1.34–4.34), iatrogenic preterm birth (OR 1.67; 95% CI 1.05–2.67), and NICU admission (OR 1.66; 95% CI 1.06–2.61) (Figure 3). There was no statistically significant difference noted with early severe ICP and other adverse outcomes (Table 4).

**TABLE 3:** Maternal baseline demographics and characteristics among early severe versus late severe ICP (sub‐analysis).

	Early severe ICP (*n* = 108)	Late severe ICP (*n* = 347)	*p* value
Gestational age at diagnosis	27.70 ± 5.12	35.93 ± 1.93	< 0.01
Peak Total Bile Acids	92.29 ± 53.62	83.97 ± 48.24	0.13
ICP Severity Cohort			0.35
Severe ICP (TBA 40‐99 µmol/L)	74 (68.52)	253 (72.91)	
Very Severe ICP (TBA ≥ 100 µmol/L)	34 (31.48)	94 (27.09)	
Age	27.95 ± 5.62	28.40 ± 5.71	0.47
Ethnicity			0.36
Hispanic	93 (86.92)	310 (89.86)	
Bengali	6 (5.61)	17 (4.93)	
European	0 (0.00)	0 (0.00)	
African	1 (0.93)	0 (0.00)	
Other/Unknown	4 (3.74)	7 (2.03)	
SE Asian	3 (2.80)	11 (3.19)	
Parity, quartiles	1 [0,2]	1 [0,2]	0.92
History of cholestasis	27 (25.00)	53 (15.32)	< 0.01
Prior preterm birth	16 (14.81)	27 (7.78)	0.03
Prior cesarean section	12 (11.54)	45 (13.55)	0.60
Prior IUFD	2 (8.70)	0 (0.00)	0.02
Pre‐gestational diabetes	2 (1.85)	5 (1.45)	0.77
Gestational diabetes	15 (13.89)	54 (15.56)	0.67
Pregnancy Induced Hypertension	7 (6.48)	42 (12.10)	0.10
Gestational age at delivery	35.95 ± 1.82	37.24 ± 1.33	< 0.01
Ursodeoxycholic acid Therapy	93 (86.11)	140 (40.35)	< 0.01
Delivery Mode			0.22
Vaginal	79 (73.15)	246 (70.89)	
Cesarean	29 (26.85)	93 (26.80)	

*Note*: All categorical variables reported as *n* (%). All continuous variables reported as mean ± SD unless listed otherwise.

**TABLE 4:** Univariable analysis of early versus late severe ICP diagnosis and adverse outcomes (sub‐analysis).

	Early severe ICP (*n* = 108)	Late severe ICP (*n* = 347)	*p* value
Spontaneous Preterm Labor	24 (22.22)	35 (10.09)	< 0.01
Umbilical Arterial pH < 7.20	7 (6.48)	28 (8.07)	0.59
Iatrogenic Preterm Birth	41 (37.96)	87 (25.07)	< 0.01
Cesarean Delivery for NRFHT	9 (8.33)	22 (6.34)	0.47
Meconium‐stained fluid	16 (14.81)	64 (18.44)	0.39
NICU admission	61 (57.55)	148 (43.92)	0.01

*Note*: All categorical variables reported as *n* (%).

Table S1 presents a univariable analysis on adverse outcomes associated with very early IHCP.

## 4 DISCUSSION

Our primary outcome analysis reveals that early diagnosis of ICP is associated with increased rates of spontaneous preterm birth. Our secondary outcome analysis reveals that early diagnosis of ICP is also associated with iatrogenic preterm birth and NICU admission. Our sub‐analysis of early severe ICP revealed similar associations, suggesting that our findings were not driven entirely by peak TBA levels. There was no association seen in the primary or sub‐analysis for early ICP and meconium‐stained amniotic fluid, CD for NRFHT, or umbilical artery pH changes.

Although recent studies have characterized the relationship between ICP severity and adverse outcomes, few studies have provided insight into outcomes with early onset ICP. As such, the clinical implication of a second or early third trimester diagnosis of ICP remains poorly understood. One prior study revealed an association of early diagnosis of ICP with higher TBA levels, and increased rates of meconium‐stained amniotic fluid, fetal distress, and neonatal asphyxia [14]. Our findings here suggest no association between early ICP and meconium‐stained amniotic fluid, but early ICP is associated with spontaneous preterm birth, iatrogenic preterm birth, and NICU admission.

Spontaneous preterm birth was not an outcome analyzed by prior studies on early ICP but has been shown to be associated with ICP [9, 10, 11]. Given the current Society for Maternal‐Fetal Medicine guidelines for iatrogenic planned delivery with severe ICP, it is not unexpected to see an association between early ICP and iatrogenic preterm birth. However, given that the association was seen even after adjusting for peak TBA levels and is also seen within our early severe ICP sub‐analysis where TBA levels did not differ, increased rates of iatrogenic preterm birth suggest possible provider discomfort with earlier diagnosis of ICP, resulting in earlier planned delivery. Owing to the combination of increased spontaneous preterm birth and iatrogenic preterm birth seen with early ICP, gestational age at delivery for early ICP was significantly lower. At our institution, all neonates less than 37 weeks’ gestation are admitted to the NICU. In the setting of this policy, our finding pertaining to NICU admission rates was not surprising but is unlikely generalizable to all settings.

Given prior studies have shown that adverse outcomes correlate with TBA levels, our findings need to be interpreted with caution since peak TBA levels in early ICP were significantly higher than late ICP [9, 10, 12]. We adjusted for peak TBA levels in our multivariable analysis, which made our strength of association less pronounced suggesting peak TBA was confounding our findings. To further mitigate this, we performed a sub‐analysis on early versus late severe ICP that did not have difference in peak TBA levels and similar associations were appreciated strengthening our conclusion. Thus, although peak TBA levels affect these adverse outcomes, our results were not driven by severity of ICP.

Patients with early diagnosis of ICP should be counseled on the risk of preterm birth. This relationship between ICP and spontaneous preterm birth is well documented in the literature, but the mechanism is not well defined [9, 10, 11]. Prior studies have shown that bile acids increase expression of oxytocin receptors and that patients with ICP may have more oxytocin sensitivity [21]. Increased duration of exposure to elevated bile acids from an earlier ICP diagnosis may result in myometrial remodeling to increase oxytocin receptors and sensitivity. Subsequently, baseline levels of circulating oxytocin that would normally have no clinical effect may trigger the onset of preterm labor in these patients. With respect to the relationship between ICP and spontaneous preterm birth now documented both with early ICP and more severe ICP by peak TBA, larger cohort studies are needed to better understand this relationship.

Additionally, prolonged fetal exposure to elevated TBA levels does not appear to confer any significant risk to the fetus or neonate. Our findings suggested that early onset ICP is not associated with meconium‐stained amniotic fluid, umbilical cord pH abnormalities, or CD for NRFHT, although it may be possible that we are underpowered to show a statistical difference in some of these outcomes. Given that the cohort only included live gestations, we are unable to comment on whether early onset ICP is associated with increased rates of stillbirth. From a maternal or fetal perspective, our findings do not suggest that altered management is warranted in the setting of early onset ICP.

The management of early onset ICP should depend on the peak TBA level reached during the pregnancy. Currently, there are no recommendations on whether or how often to re‐test TBA levels and whether or how to alter management with subsequent TBA levels. Although some studies have assessed serial TBA levels in pregnancy, future studies are needed to determine better characterize TBA trends with ICP, especially in the setting of early diagnosis [22, 23, 24]. We often empirically recheck TBA levels every 3–4 weeks and base delivery timing on the peak TBA level reached. However, we acknowledge that no set guidelines or protocols have been well studied.

Our study has several limitations. Despite the consistent management and size of our ICP cohort, we are still underpowered to perform some key analyses. To better characterize the effects of early ICP, it would be pertinent to perform a sub‐analysis of very early onset of ICP (< 28‐week gestation) to those with later onset (> 32‐week gestation). Although we have supplied descriptive data on very early ICP in Table S1, our sample size limits our ability to perform an appropriate analysis of this degree. Despite similar baseline characteristics, as in any retrospective study, there remains the possibility of confounding or bias from unmeasured variables. Additionally, although prior early ICP studies were underpowered to assess for stillbirth, we are limited in that our cohort only includes live gestations, and thus, we are unable to analyze stillbirth risk. Recently, there has been ongoing debates for re‐defining ICP diagnostic thresholds but our study is based on current diagnostic cutoff of TBA ≥ 10 µmol/L [25, 26, 27]. Despite the previously mentioned advantages of consistent management provided through a single institution, our high‐prevalence setting limits the generalizability of our study. With respect to institutional management, our planned NICU admission for all preterm neonates limits the generalizability of our admission to NICU outcome, however, given the changes in electronic medical record software and the secondary analysis nature of this study, we are unable to comment on indications for NICU admission at this time. Finally, since our patients were not fasting at the time of TBA blood testing, this may have resulted in an overdiagnosis of ICP [28].

Our study also has multiple strengths. Prior ICP studies have been limited by smaller cohorts and thus a relative inability to adequately perform studies on exposures such as early diagnosis of ICP. Additionally, our entire cohort received all prenatal and delivered at a single institution, resulting in consistent management. Since one electronic medical record was utilized, this resulted in complete medical records for the variables of interest. During our study timeline from 2005 to 2019, the most significant ICP management change came in 2019 with the finding that stillbirth rates correlate positively with ICP severity [9]. Since our cohort ends in 2019, the potential bias introduced by these advancements are limited. Finally, our cohorts were relatively similar from a baseline demographics perspective.

## 5 CONCLUSION

Our study findings suggest that early diagnosis of ICP at less than 32‐week gestation is associated with increased odds of adverse outcomes, including spontaneous preterm birth, iatrogenic preterm birth, and NICU admission. Given the findings are consistent even when sub‐analyzing early severe ICP, we believe these adverse outcomes are not entirely dependent on ICP severity but also on the duration of time exposed to elevated bile acids. While this study better describes the outcomes associated with cholestasis, further studies are needed to determine the mechanism for early ICP associated spontaneous preterm birth and whether trending TBA after early diagnosis can improve outcomes.

## CONFLICT OF INTEREST STATEMENT

The authors declare no conflicts of interest.

## DISCLOSURES

The primary retrospective cohort study was approved by the Institutional Review Board at the Icahn School of Medicine at Mount Sinai prior to accessing the electronic medical record. No component of this original manuscript has previously been published elsewhere and is not under consideration for publication in any journal aside from the *Pregnancy* Journal.

## Supporting information



Supporting Information

## Data Availability

The data that support the findings of this study are available from the corresponding author upon reasonable request.
