# Supplementary material for: Earlier diagnosis of intrahepatic cholestasis of pregnancy and adverse pregnancy outcomes
Source: Pregnancy (Hoboken). 2025 Jul 16;1(4):e70073. doi: 10.1002/pmf2.70073 (PMC12435465; doi:10.1002/pmf2.70073)
Supplement: Supplementary file 1 [file PMF2-1-e70073-s001.docx]

| **Supplemental Table 1. Univariable Analysis of Very Early (less than 28-week diagnosis) vs not-Very Early ICP diagnosis and adverse outcomes (sensitivity analysis)**  - All categorical variables reported as n (%) | | | |
| --- | --- | --- | --- |
|  | **Very Early ICP (n=85)** | **Not-Very Early ICP (n=1162)** | **p-value** |
| **Spontaneous Preterm Labor** | 11 (12.94) | 84 (7.23) | 0.06 |
| **Umbilical Arterial pH <7.20** | 4 (4.71) | 92 (7.92) | 0.28 |
| **Iatrogenic Preterm Birth** | 23 (27.06) | 203 (17.47) | 0.03 |
| **Cesarean Delivery for Non-Reassuring Fetal Heart Rate Tracing** | 13 (15.29) | 78 (6.71) | <0.01 |
| **Meconium-stained fluid** | 8 (9.41) | 135 (11.62) | 0.54 |
| **Neonatal ICU admission** | 36 (45.00) | 438 (39.25) | 0.31 |
